# Characterizations of Some Fuzzy Prefilters (Filters) in *EQ*-Algebras

**DOI:** 10.1155/2014/829527

**Published:** 2014-05-05

**Authors:** Xiao Long Xin, Peng Fei He, Yong Wei Yang

**Affiliations:** Department of Mathematics, Northwest University, Xi'an 710127, China

## Abstract

We introduce and study some types of fuzzy prefilters (filters) in *EQ*-algebras. First, we present several characterizations of fuzzy positive implicative prefilters (filters), fuzzy implicative prefilters (filters), and fuzzy fantastic prefilters (filters). Next, using their characterizations, we mainly consider the relationships among these special fuzzy filters. Particularly, we find some conditions under which a fuzzy implicative prefilter (filter) is equivalent to a fuzzy positive implicative prefilter (filter). As applications, we obtain some new results about classical filters in *EQ*-algebras and some related results about fuzzy filters in residuated lattices.

## 1. Introduction

Every many-valued logic is uniquely determined by the algebraic properties of the structure of its truth values. At present, it is generally accepted that, in fuzzy logic, the algebraic structure should be a residuated lattice, possibly fulfilling some additional properties. *BL*-algebras, *MTL*-algebras, *MV*-algebras, and so forth are the best known classes of residuated lattices [1–3]. Note that the typical operations on these algebras are multiplication ⊙ and implication → which are closely tied by adjointness property.

Fuzzy type theory [4, 5] whose basic connective is a fuzzy equality was developed as a counterpart of the classical higher-order logic (type theory in which identity is a basic connective; see [6]). Since the algebra of truth values is no longer a residuated lattice, a specific algebra called an *EQ*-algebra [7] for fuzzy type theory was proposed by Novák and De Baets. *EQ*-algebras are interesting and important algebras from many points of view. First, the above residuated lattices based logical algebras are all particular cases of *EQ*-algebras. Second, the adjointness property which strictly couples ⊙ and → on residuated lattices based logical algebras is relaxed. Indeed, in *EQ*-algebras, → is defined directly from fuzzy equality by the formula *x* → *y* = (*x*∧*y*) ~ *x*. But the fuzzy equality ~ cannot be reconstructed from the implication → in *EQ*-algebras in general. Third, *EQ*-algebras open a possibility to develop a fuzzy logic with a noncommutative conjunction but a single implication only [7]. From these points of view, it is meaningful to study *EQ*-algebras.

The filter theory plays an important role in studying logical algebras. From logic point of view, various filters have natural interpretation as various sets of provable formulas. Up to now, some types of filters on special residuated lattices based logical algebras have been widely studied and some important results are obtained [8–12]. It is proved that Boolean filters and implicative filters coincide together in *BL*-algebras [11, 13], and that implicative filters are positive implicative filters in *BL*-algebras, but they all are equivalent in *MV*-algebras [8, 14]. Moreover, the properties of filters have a strong influence on the structure properties of algebras.

The sets of provable formulas in corresponding inference systems from the point of view of uncertain information can be described by fuzzy filters of those algebraic semantics. At present, many authors studied some kinds of fuzzy filters of various logics algebras [8, 9, 13–19]. Liu and Li [13–15] introduced and studied the fuzzy filters, fuzzy implicative filters, fuzzy Boolean filters, and fuzzy positive implicative filters in *BL*-algebras and *R*_0_-algebras, respectively. Ma et al. established generalized fuzzy filter theory of *BL*-algebras in [20–23]. Recently, Zhang et al. have extended the notions of special fuzzy filters to general residuated lattices [24, 25].

Since residuated lattices (*BL*-algebras, *MV*-algebras, *MTL*-algebras, and *R*_0_-algebras) are particular types of *EQ*-algebras, it is natural and meaningful to extend some notions of residuated lattices to *EQ*-algebras and establish more general theories in *EQ*-algebras for studying the common properties of the abovementioned algebras. For *EQ*-algebras, the notions of prefilters (which coincide with filters in residuated lattices) and prime prefilters were proposed and some of their properties were obtained [26]. In [27], implicative and positive implicative prefilters of *EQ*-algebras have been studied and some interesting results have been obtained.

The aim of this paper is to characterize some types of fuzzy prefilters (filters) and to discuss the relationships among these notions in *EQ*-algebras. Moreover, using our obtained results about various fuzzy filters in *EQ*-algebras, we further develop the classical filter theory in *EQ*-algebras and the fuzzy filter theory in residuated lattices. In this paper, several characterizations of fuzzy positive implicative prefilters (filters), fuzzy implicative prefilters (filters), and fuzzy fantastic prefilters (filters) in *EQ*-algebras are derived. Moreover, the relations among these fuzzy prefilters (filters) are considered. As applications of our obtained results, we give some new results about classical filters in *EQ*-algebras and some related results about fuzzy filters in residuated lattices. Based on the results obtained in this paper, it will lay a foundation for providing an algebraic tool in considering many problems in fuzzy logic.

## 2. Preliminaries

In the section, we present some definitions and results about *EQ*-algebras that will be used in the sequel.

**Definition 1 (see [7, 26])**

An algebra (*L*, ∧, ⊙, ~, 1) of type (2,2, 2,0) is called an *EQ*-algebra if it satisfies the following axioms:

(*E*1) (*L*, ∧, 1) is a ∧-semilattice with top element 1. We set *x* ≤ *y* if and only if *x*∧*y* = *x*, as usual;
(*E*2) (*L*, ⊙, 1) is a commutative monoid and ⊙ is isotone with respect to ≤;
(*E*3) *x* ~ *x* = 1 (reflexivity axiom);
(*E*4) ((*x*∧*y*) ~ *z*)⊙(*s* ~ *x*) ≤ *z* ~ (*s*∧*y*) (substitution axiom);
(*E*5) (*x* ~ *y*)⊙(*s* ~ *t*)≤(*x* ~ *s*)~(*y* ~ *t*) (congruence axiom);
(*E*6) (*x*∧*y*∧*z*) ~ *x* ≤ (*x*∧*y*) ~ *x* (monotonicity axiom);
(*E*7) *x*⊙*y* ≤ *x* ~ *y* (boundedness axiom), for all *x*, *y*, *z*, *s*, *t* ∈ *L*.

Let *L* be an *EQ*-algebra. For all *x* ∈ *L*, we put $\tilde{x}=x\sim 1$. If *L* contains a bottom element 0, then we may define the unary operation ¬ on *L* by ¬*x* = *x* ~ 0.

**Definition 2 (see [7])**

An *EQ*-algebra *L* is called

1. a* good* *EQ*-algebra if $\tilde{x}=x$ for all *x* ∈ *L*,
2. a* separated* *EQ*-algebra if *x* ~ *y* = 1 implies that *x* = *y* for all *x*, *y* ∈ *L*,
3. a* residuated* *EQ*-algebra if (*x*⊙*y*)∧*z* = *x*⊙*y* if and only if *x*∧((*y*∧*z*) ~ *y*) = *x* for all *x*, *y*, *z* ∈ *L*,
4. an* involutive* *EQ*-algebra if it contains a bottom element 0 and for all *x* ∈ *L* it holds that ¬¬*x* = *x*.

Let *L* be an *EQ*-algebra. For all *x*, *y* ∈ *L*, we put *x* → *y* = (*x*∧*y*) ~ *x*.

The derived operation → is called an* implication*.

**Remark 3**

Let (*L*, ∧, ∨, ⊙, ⇒, 0,1) be a residuated lattice. For any *x*, *y* ∈ *L*, we define *x* ~ *y* = (*x*⇒*y*)∧(*y*⇒*x*). Then, (*L*, ∧, ⊙, ~, 1) is a residuated *EQ*-algebra [7]. It is easily proved that *x* → *y* = (*x*∧*y*) ~ *x* = *x*⇒*y*. In general, a residuated *EQ*-algebra may not be a residuated lattice [26]. This shows that residuated lattices are proper classes of *EQ*-algebras [27].

The following properties are well known to hold in *EQ*-algebras, we summarize them as follows.

**Lemma 4 (see [7, 26])**

Let (*L*, ∧, ⊙, ~, 1) be an *EQ*-algebra. Then, the following properties hold:

1. *x* ~ *y* = *y* ~ *x*, *x* ~ *y* ≤ *x* → *y*,
2. *x* ≤ 1 ~ *x* = 1 → *x* ≤ *y* → *x*,
3. *x*⊙*y* ≤ *x*∧*y* ≤ *x*, *y*,
4. (*x* ~ *y*)⊙(*y* ~ *z*) ≤ *x* ~ *z*,
5. (*x* → *y*)⊙(*y* → *z*) ≤ *x* → *z*,
6. *x* ~ *y* ≤ (*x* ~ *z*)~(*y* ~ *z*),
7. *x* → *y* ≤ (*y* → *z*)→(*x* → *z*),
8. *x* → *y* ≤ (*z* → *x*)→(*z* → *y*),
9. *x* ~ *y* ≤ (*x*∧*z*)~(*y*∧*z*),
10. *x* → *y* ≤ (*x*∧*z*)→(*y*∧*z*),
11. *x* → *y* = *x* → (*x*∧*y*),
12. if *x* ≤ *y*, then *x* → *y* = 1, *x* ~ *y* = *y* → *x*,
13. if *x* ≤ *y*, then *y* → *z* ≤ *x* → *z*, *z* → *x* ≤ *z* → *y*,
14. if *L* contains a bottom element 0, then ¬0 = 1, ¬*x* = *x* → 0,
15. if *L* is a residuated *EQ*-algebra, then *x*⊙*y* → *z* ≤ *x* → (*y* → *z*), for any *x*, *y*, *z* ∈ *L*.

**Lemma 5 (see [7, 26])**

Let *L* be a good *EQ*-algebra. Then, the following properties hold, for any *x*, *y*, *z* ∈ *L*:

1. *x* ≤ (*x* → *y*) → *y*,
2. *x* → (*y* → *z*) = *y* → (*x* → *z*),
3. *x*⊙(*x* → *y*) ≤ *y*,
4. *x*⊙(*x* → *y*) ≤ *x*∧*y*.

**Lemma 6 (see [26])**

Let *L* be an *EQ*-algebra. Then, the following are equivalent:

1. an *EQ*-algebra *L* is residuated;
2. the *EQ*-algebra *L* is good and *x* → *y* ≤ (*x*⊙*z*)→(*y*⊙*z*) for any *x*, *y*, *z* ∈ *L*;
3. the *EQ*-algebra *L* is good and *x* ≤ *y* → (*x*⊙*y*) for any *x*, *y* ∈ *L*.

In what follows, we recall the notions of some prefilters (filters) in *EQ*-algebras.

**Definition 7 (see [26])**

A nonempty subset *F* of an *EQ*-algebra *L* is called a* prefilter* of *L* if it satisfies, for any *x*, *y* ∈ *L*,

(F1) 1 ∈ *F*,
(F2) *x* ∈ *F*; *x* → *y* ∈ *F* imply that *y* ∈ *F*,
 a prefilter *F* is called a* filter* if it satisfies, for any *x*, *y*, *z* ∈ *L*,
(F3) *x* → *y* ∈ *F* imply (*x*⊙*z*)→(*y*⊙*z*) ∈ *F*.

A prefilter *F* of an *EQ*-algebra *L* is called* proper* if and only if *F* ≠ *L*.

**Definition 8 (see [27])**

A prefilter *F* of an *EQ*-algebra *L* is called a* positive implicative prefilter* of *L* if it satisfies, for any *x*, *y*, *z* ∈ *L*,

(F4) if *x* → (*y* → *z*) ∈ *F* and *x* → *y* ∈ *F*, then *x* → *z* ∈ *F*.

If *F* is a filter and it satisfies (F4), then *F* is called a* positive implicative filter* of *L*.

**Definition 9 (see [27])**

A nonempty subset *F* of an *EQ*-algebra *L* is called an* implicative prefilter* of *L* if it satisfies (F1) and

(F5) *z* → ((*x* → *y*) → *x*) ∈ *F* and *z* ∈ *F* imply that *x* ∈ *F* for any *x*, *y*, *z* ∈ *L*.

An implicative prefilter *F* is called an* implicative filter* if it satisfies (F3).

Now, we review the concept of fuzzy sets; see [28].

A* fuzzy set* in *L* is a mapping *μ*: *L* → [0,1].

Let *μ* be a fuzzy set in *L*. For all *t* ∈ [0,1], the set *μ*_*t*_ = {*x* ∈ *L* | *μ*(*x*) ≥ *t*} is called a* level subset* of *μ*.

Let *F* be a nonempty subset, we denote the characteristic function of *F* by *χ*_*F*_.

For convenience, for any *a*, *b* ∈ [0,1], we denote max⁡{*a*, *b*} and min⁡{*a*, *b*} by *a*∨*b* and *a*∧*b*, respectively.

For any fuzzy sets *μ*, *ν* in *L*, we define *μ*⊆*ν* if and only if *μ*(*x*) ≤ *ν*(*x*) for all *x* ∈ *L*.

## 3. Fuzzy Prefilters (Filters) in *EQ*-Algebras

In this section, we introduce the notion of fuzzy prefilters (filters) in *EQ*-algebras and give some of their properties that will be used in the sequel.

In what follows, let *L* denote an *EQ*-algebra unless otherwise is specified.

**Definition 10**

Let *μ* be a fuzzy set in *L*. *μ* is called a* fuzzy prefilter* of *L* if it satisfies

(FF1) *μ*(1) ≥ *μ*(*x*) for all *x* ∈ *L*,
(FF2) *μ*(*y*) ≥ *μ*(*x*)∧*μ*(*x* → *y*) for all *x*, *y* ∈ *L*.
 A fuzzy prefilter *μ* is called a* fuzzy filter* if it satisfies
(FF3) *μ*((*x*⊙*z*)→(*y*⊙*z*)) ≥ *μ*(*x* → *y*) for all *x*, *y*, *z* ∈ *L*.

The following examples show that fuzzy prefilters (filters) in *EQ*-algebras exist.

**Example 11**

Let *L* = {0, *a*, *b*, 1} be a chain with Cayley tables as Table 1

Then, (*L*, ∧, ⊙, ~, 1) is an *EQ*-algebra in [27]. Define a fuzzy set *μ* in *L* as follows: *μ*(1) = 0.8, *μ*(*b*) = 0.6, and *μ*(0) = *μ*(*a*) = 0.4. One can check that *μ* is a fuzzy prefilter of *L*.

**Example 12**

Let *L* = {0, *a*, *b*, 1} be a chain with Cayley tables as Table 2.

Then, (*L*, ∧, ⊙, ~, 1) is an *EQ*-algebra. Define a fuzzy set *μ* in *L* as follows: *μ*(1) = *μ*(*b*) = 0.8 and *μ*(0) = *μ*(*a*) = 0.4. It is easy to check that *μ* is a fuzzy filter of *L*.

**Proposition 13**

Let *μ* be a fuzzy prefilter of *L*. For all *x*, *y* ∈ *L*, if *x* ≤ *y*, then *μ*(*x*) ≤ *μ*(*y*); that is, *μ* is order-preserving.

**Proof**

It is easy and omitted.

**Remark 14**

From Lemma 6, in a residuated *EQ*-algebra, we know that *x* → *y* ≤ (*x*⊙*z*)→(*y*⊙*z*) for any *x*, *y*, *z* ∈ *L*. Combining Proposition 13, we can obtain that fuzzy prefilters and fuzzy filters coincide in residuated *EQ*-algebras.

By using the level prefilters (filters) of an *E*Q-algebra, we can characterize fuzzy prefilters (filters).

**Theorem 15**

Let *μ* be a fuzzy set in *L*. *μ* is a fuzzy prefilter (filter) of *L* if and only if, for all *t* ∈ [0,1], *μ*_*t*_ is either empty or a prefilter (filter) of *L*.

**Proof**

The  proof  is straightforward.

**Corollary 16**

Let *F* be a nonempty subset of *L*. *F* is a prefilter (filter) if and only if *χ*_*F*_ is a fuzzy prefilter (filter) of *L*.

Next, we discuss some properties of fuzzy filters in an *EQ*-algebra, which will be used in the sequel.

**Proposition 17**

Let *L* be an *EQ*-algebra and let *μ* be a fuzzy filter of *L*; for all *x*, *y*, *z* ∈ *L*, the following hold:

1. *μ*(*x*⊙*y*) = *μ*(*x*)∧*μ*(*y*),
2. *μ*(*x* → *z*) ≥ *μ*(*x* → *y*)∧*μ*(*y* → *z*).

**Proof**

(1) Since *x*⊙*y* ≤ *x*∧*y* ≤ *x*, *y*, then *μ*(*x*⊙*y*) ≤ *μ*(*x*)∧*μ*(*y*) by Proposition 13. From *y* ≤ 1 → *y*, it follows that *μ*(*y*) ≤ *μ*(1 → *y*). Since *μ* is a fuzzy filter of *L*, then *μ*((*x*⊙1)→(*x*⊙*y*)) ≥ *μ*(1 → *y*) by (FF3); that is, *μ*(*x* → (*x*⊙*y*)) ≥ *μ*(1 → *y*). By (FF2), we get *μ*(*x*⊙*y*) ≥ *μ*(*x*)∧*μ*(*x* → (*x*⊙*y*)). Hence, we have *μ*(*x*⊙*y*) ≥ *μ*(*x*)∧*μ*(*x* → (*x*⊙*y*)) ≥ *μ*(*x*)∧*μ*(1 → *y*) ≥ *μ*(*x*)∧*μ*(*y*). Therefore, *μ*(*x*⊙*y*) = *μ*(*x*)∧*μ*(*y*).

(2) Since (*x* → *y*)⊙(*y* → *z*) ≤ *x* → *z*, we have *μ*(*x* → *z*) ≥ *μ*((*x* → *y*)⊙(*y* → *z*)) by Proposition 13. From (1), it follows that *μ*((*x* → *y*)⊙(*y* → *z*)) = *μ*(*x* → *y*)∧*μ*(*y* → *z*). Therefore, *μ*(*x* → *z*) ≥ *μ*(*x* → *y*)∧*μ*(*y* → *z*).

## 4. Fuzzy Positive Implicative Prefilters (Filters)

In the section, we introduce the notion of fuzzy positive implicative prefilters (filters) in *EQ*-algebras and give some of their characterizations. Moreover, applying these characterizations, we obtain some new results about positive implicative filters in *EQ*-algebras.

**Definition 18**

Let *μ* be a fuzzy prefilter in *L*. *μ* is called a* fuzzy positive implicative prefilter* of *L* if it satisfies

(FF4) *μ*(*x* → *z*) ≥ *μ*(*x* → (*y* → *z*))∧*μ*(*x* → *y*) for all *x*, *y*, *z* ∈ *L*.

A fuzzy filter *μ* of *L* is called a* fuzzy positive implicative filter* if it satisfies (FF4).

For better understanding of the above definition, we illustrate it by the following example.

**Example 19**

Let *L* be the *EQ*-algebra and let *μ* be the fuzzy set of *L* defined in Example 12. One can see that *μ* is a fuzzy positive implicative filter of *L*.

The following result gives a characterization of fuzzy positive implicative prefilters by fuzzy prefilters.

**Theorem 20**

Let *μ* be a fuzzy prefilter in *L*. *μ* is a fuzzy positive implicative prefilter of *L* if and only if *μ* ^*a*^ : *L* → [0,1] is a fuzzy prefilter in *L*, where *μ* ^*a*^(*x*) = *μ*(*a* → *x*) for all *a*, *x* ∈ *L*.

**Proof**

Suppose  that  *μ* is a fuzzy positive implicative prefilter of *L*. Since *a* → 1 = 1, then *μ*(*a* → 1) = *μ*(1). It follows that *μ* ^*a*^(1) = *μ*(*a* → 1) = *μ*(1). From *a* → *x* ≤ 1, we have *μ*(*a* → *x*) ≤ *μ*(1); that is, *μ* ^*a*^(*x*) ≤ *μ*(1). Then, *μ* ^*a*^(*x*) ≤ *μ* ^*a*^(1). On the other hand, since *μ* is a fuzzy positive implicative prefilter of *L*, then *μ*(*a* → *y*) ≥ *μ*(*a* → (*x* → *y*))∧*μ*(*a* → *x*); that is, *μ* ^*a*^(*y*) ≥ *μ* ^*a*^(*x* → *y*)∧*μ* ^*a*^(*x*). Therefore, *μ* ^*a*^ is a fuzzy prefilter in *L*.

Conversely, for any *a* ∈ *L*, since *μ* ^*a*^ is a fuzzy prefilter in *L*, then *μ* ^*x*^ is a fuzzy prefilter in *L*. It follows that *μ* ^*x*^(*z*) ≥ *μ* ^*x*^(*y* → *z*)∧*μ* ^*x*^(*y*). By the definition of *μ* ^*x*^, we have *μ*(*x* → *z*) ≥ *μ*(*x* → (*y* → *z*))∧*μ*(*x* → *y*). Therefore, *μ* is a fuzzy positive implicative prefilter of *L*.

As a consequence of Theorem 20, we have the following properties of *μ* ^*a*^.

**Proposition 21**

Let *μ* be a fuzzy positive implicative prefilter of *L*. Then, for any *a* ∈ *L*, *μ* ^*a*^ is the fuzzy prefilter containing *μ*.

**Proof**

Assume that *μ* is a fuzzy  positive implicative prefilter of *L*. By Theorem 20, we obtain that *μ* ^*a*^ is a fuzzy prefilter. For any *x* ∈ *L*, *x* ≤ *a* → *x*. It follows from Proposition 13 that *μ*(*x*) ≤ *μ*(*a* → *x*); that is, *μ*(*x*) ≤ *μ* ^*a*^(*x*). And so *μ*⊆*μ* ^*a*^. Therefore, *μ* ^*a*^ is the fuzzy prefilter containing *μ*.

**Proposition 22**

Let *μ*, *ν* be two fuzzy prefilters of *L*. Then, for any *a*, *b* ∈ *L*, the following statements hold:

1. *μ* ^*a*^ = *μ* if and only if *μ*(*a*) = *μ*(1),
2. *a* ≤ *b* implies that *μ* ^*b*^⊆*μ* ^*a*^,
3. *μ*⊆*ν* implies that *μ* ^*a*^⊆*ν* ^*a*^,
4. (*μ*∩*ν*)^*a*^ = *μ* ^*a*^∩*ν* ^*a*^, (*μ* ∪ *ν*)^*a*^ = *μ* ^*a*^ ∪ *ν* ^*a*^.

**Proof**

(1) Suppose that *μ* ^*a*^ = *μ*. Then, *μ*(*a*) = *μ* ^*a*^(*a*) = *μ*(*a* → *a*) = *μ*(1); hence, *μ*(*a*) = *μ*(1).

Conversely, assume that *μ*(*a*) = *μ*(1). For any *x* ∈ *I*, since *x* ≤ *a* → *x* and *μ* is a fuzzy prefilter, we have *μ*(*x*) ≤ *μ*(*a* → *x*) = *μ* ^*a*^(*x*). Hence, *μ*⊆*μ* ^*a*^. On the other hand, since *μ* is a fuzzy prefilter, then *μ*(*x*) ≥ *μ*(*a* → *x*)∧*μ*(*a*) = *μ*(*a* → *x*)∧*μ*(1) = *μ*(*a* → *x*) for all *x* ∈ *L*. It follows that *μ*(*x*) ≥ *μ* ^*a*^(*x*); that is, *μ*⊇*μ* ^*a*^. Consequently, we obtain *μ* ^*a*^ = *μ*.

(2) Suppose that *a* ≤ *b*. For any *x* ∈ *L*, then *b* → *x* ≤ *a* → *x*. Since *μ* is a fuzzy prefilter, we have *μ*(*b* → *x*) ≤ *μ*(*a* → *x*). It follows that *μ* ^b^(*x*) ≤ *μ* ^*a*^(*x*). So *μ* ^*b*^⊆*μ* ^*a*^.

(3) and (4) It is easy to prove them, and we hence omit the details.

In the following, we give some equivalent conditions of fuzzy positive implicative prefilters (filters) for further discussions.

**Theorem 23**

Let *μ* be a fuzzy prefilter (filter) in *L*. The following are equivalent:

1. *μ* is a fuzzy positive implicative prefilter (filter) of *L*,
2. *μ*(*x* → *y*) ≥ *μ*(*x* → (*x* → *y*)) for all *x*, *y* ∈ *L*,
3. *μ*(*x* → *y*) = *μ*(*x* → (*x* → *y*)) for all *x*, *y* ∈ *L*.

**Proof**

  (1)⇒(2)  Suppose  that *μ* is a fuzzy positive implicative prefilter of *L*. Then, *μ*(*x* → *y*) ≥ *μ*(*x* → (*x* → *y*))∧*μ*(*x* → *x*) follows from Definition 18. Consequently, we have *μ*(*x* → *y*) ≥ *μ*(*x* → (*x* → *y*)).

(2)⇒(3) From *x* → *y* ≤ *x* → (*x* → *y*), it follows that *μ*(*x* → *y*) ≤ *μ*(*x* → (*x* → *y*)), which together with (2) leads to *μ*(*x* → *y*) = *μ*(*x* → (*x* → *y*)).

(3)⇒(1) By Lemma 4 (7), we have *x* → (*y* → *z*)≤((*y* → *z*)→(*x* → *z*))→(*x* → (*x* → *z*)) and *x* → *y* ≤ (*y* → *z*)→(*x* → *z*). Since *μ* is a fuzzy prefilter in *L*, then *μ*(*x* → (*y* → *z*)) ≤ *μ*(((*y* → *z*)→(*x* → *z*))→(*x* → (*x* → *z*))) and *μ*(*x* → *y*) ≤ *μ*((*y* → *z*)→(*x* → *z*)). From Definition 10, it follows that *μ*(*x* → (*x* → *z*)) ≥ *μ*((*y* → *z*)→(*x* → *z*))∧*μ*((*y* → *z*)→(*x* → *z*))→(*x* → (*x* → *z*))) ≥ *μ*(*x* → *y*)∧*μ*(*x* → (*y* → *z*)). Combining (3), we get *μ*(*x* → *z*) ≥ *μ*(*x* → *y*)∧*μ*(*x* → (*y* → *z*)). Therefore, *μ* is a fuzzy positive implicative prefilter of *L*.

**Theorem 24**

Let *μ* be a fuzzy set in *L*. *μ* is a fuzzy positive implicative prefilter (filter) of *L* if and only if, for all *t* ∈ [0,1], *μ*_*t*_ is either empty or a positive implicative prefilter (filter) of *L*.

**Proof**

The proof  is easy, and we hence omit the details.

**Corollary 25**

Let *F* be a nonempty subset of *L*. *F* is a positive implicative prefilter (filter) if and only if *χ*_*F*_ is a fuzzy positive implicative prefilter (filter).

Now, we continue to characterize fuzzy positive implicative filters.

**Theorem 26**

Let *L* be an *EQ*-algebra and let *μ* be a fuzzy filter. Then, *μ* is a fuzzy positive implicative filter of *L* if and only if *μ*(*x*∧(*x* → *y*) → *y*) = *μ*(1) for all *x*, *y* ∈ *L*.

**Proof**

Since *μ* is a fuzzy positive implicative filter of *L*, then *μ*(*x*∧  (*x* → *y*) → *y*) ≥ *μ*(*x*∧(*x* → *y*)→(*x* → *y*))  ∧  *μ*(*x*∧(*x* → *y*) → *x*). For any *x*, *y* ∈ *L*, since *x*∧(*x* → *y*) ≤ *x* → *y* and *x*∧(*x* → *y*) ≤ *x*, then *x*∧(*x* → *y*)→(*x* → *y*) = 1 and *x*∧(*x* → *y*) → *x* = 1. Hence, *μ*(*x*∧(*x* → *y*)→(*x* → *y*)) = *μ*(1) and *μ*(*x*∧(*x* → *y*) → *x*) = *μ*(1). Consequently, we have *μ*(*x*∧(*x* → *y*) → *y*) = *μ*(1).

Conversely, by Proposition 17, we have *μ*(*x* → *z*) ≥ *μ*(*x* → (*y*∧(*y* → *z*)))∧*μ*((*y*∧(*y* → *z*)) → *z*) ≥ *μ*(*x* → (*x*∧*y*))∧  *μ*((*x*∧*y*)→(*y*∧  (*y* → *z*)))  ∧  *μ*(*y*∧  (*y* → *z*) → *z*). It follows from Lemma 4 that *x* → (*y* → *z*)≤(*x*∧*y*)→(*y*∧(*y* → *z*)) and *x* → *y* = *x* → (*x*∧  *y*). Then, *μ*((*x*∧*y*)→(*y*∧(*y* → *z*))) ≥ *μ*(*x* → (*y* → *z*)) and *μ*(*x* → (*x*∧*y*)) = *μ*(*x* → *y*). Combining *μ*(*y*∧(*y* → *z*) → *z*) = *μ*(1), we obtain that *μ*(*x* → *z*) ≥ *μ*(*x* → (*y* → *z*))∧*μ*(*x* → *y*). Therefore, *μ* is a fuzzy positive implicative filter of *L*.

In order to get some related results about fuzzy filters in residuated lattices (see Corollary 66), we characterize fuzzy positive implicative filters in a residuated *EQ*-algebra.

**Theorem 27**

Let *L* be a residuated *EQ*-algebra and *μ* be a fuzzy filter. The following are equivalent:

1. *μ* is a fuzzy positive implicative filter of *L*,
2. *μ*(*x* → (*x*⊙*x*)) = *μ*(1) for all *x* ∈ *L*,
3. *μ*(*x*∧*y* → (*x*⊙*y*)) = *μ*(1) for all *x*, *y* ∈ *L*,
4. *μ*(*x*∧(*x* → *y*)→(*x*⊙*y*)) = *μ*(1) for all *x*, *y* ∈ *L*.

**Proof**

  (1)⇔(2) Since *L* is a residuated *EQ*-algebra, we have 1 = (*x*⊙*x*)→(*x*⊙*x*) ≤ *x* → (*x* → (*x*⊙*x*)) by Lemma 4 (15). By Proposition 13, we get *μ*(1) ≤ *μ*(*x* → (*x* → (*x*⊙*x*))), which implies that *μ*(*x* → (*x* → (*x*⊙*x*))) = *μ*(1). From Definition 18, it follows that *μ*(*x* → (*x*⊙*x*)) ≥ *μ*(*x* → (*x* → (*x*⊙*x*)))∧*μ*(*x* → *x*) = *μ*(*x* → (*x* → (*x*⊙*x*))) = *μ*(1). Consequently, we have *μ*(*x* → (*x*⊙*x*)) = *μ*(1).

Conversely, by Proposition 17, we have *μ*(*x* → *z*) ≥ *μ*(*x* → (*x*⊙*x*))∧*μ*((*x*⊙*x*) → *z*) = *μ*(1)∧*μ*((*x*⊙*x*) → *z*) = *μ*((*x*⊙  *x*) → *z*) ≥ *μ*((*x*⊙  *x*)→(*y*⊙  (*y* → *z*)))∧*μ*((*y*⊙  (*y* → *z*)) → *z*). By Lemma 5 (3), we have *y*⊙(*y* → *z*) ≤ *z*. Then, *μ*((*y*⊙(*y* → *z*)) → *z*) = *μ*(1). It follows that *μ*(*x* → *z*) ≥ *μ*((*x*⊙*x*)→(*y*⊙(*y* → *z*))). Hence, we obtain that *μ*(*x* → *z*) ≥ *μ*((*x*⊙*x*)→(*y*⊙(*y* → *z*))) ≥ *μ*((*x*⊙  *x*)→(*x*⊙  *y*))  ∧  *μ*((*x*⊙  *y*)→(*y*⊙  (*y* → *z*))) by Proposition 17. Since *μ* is a fuzzy filter, we have *μ*((*x*⊙*x*)→(*x*⊙*y*)) ≥ *μ*(*x* → *y*) and *μ*((*x*⊙*y*)→(*y*⊙(*y* → *z*))) ≥ *μ*(*x* → (*y* → *z*)) by (FF3). Combining them, we get *μ*(*x* → *z*) ≥ *μ*(*x* → (*y* → *z*))∧*μ*(*x* → *y*). Therefore, *μ* is a fuzzy positive implicative filter of *L*.

(1)⇒(3) Suppose that *μ* is a fuzzy positive implicative filter of *L*. Then, *μ*(*x*∧(*x* → (*x*⊙*y*))→(*x*⊙*y*)) = *μ*(1) by Theorem 26. Since *L* is a residuated *EQ*-algebra, we have *y* ≤ *x* → (*x*⊙*y*) by Lemma 6 (3). Then, *x*∧*y* ≤ *x*∧(*x* → (*x*⊙*y*)), which implies that *x*∧(*x* → (*x*⊙*y*))→(*x*⊙*y*) ≤ *x*∧*y* → (*x*⊙*y*). From Proposition 13, we have *μ*(*x*∧*y* → (*x*⊙*y*)) ≥ *μ*(*x*∧(*x* → (*x*⊙*y*))→(*x*⊙*y*)) = *μ*(1). Therefore, we have *μ*(*x*∧*y* → (*x*⊙*y*)) = *μ*(1).

(3)⇒(4) From (3), it follows that *μ*(*x*∧(*x* → *y*)→(*x*⊙(*x* → *y*))) = *μ*(1). By Lemma 5, we have *x*⊙(*x* → *y*) ≤ *x*∧*y*, which implies that *x*∧(*x* → *y*)→(*x*⊙(*x* → *y*)) ≤ *x*∧(*x* → *y*)→(*x*∧*y*). Then, *μ*(*x*∧(*x* → *y*)→(*x*⊙(*x* → *y*))) ≤ *μ*(*x*∧(*x* → *y*)→(*x*∧*y*)). Hence, *μ*(*x*∧(*x* → *y*)→(*x*∧*y*)) = *μ*(1). By Proposition 17, we have *μ*(*x*∧(*x* → *y*)→(*x*⊙*y*)) ≥ *μ*(*x*∧(*x* → *y*)→(*x*∧*y*))∧*μ*(*x*∧*y* → (*x*⊙*y*)) = *μ*(1)∧*μ*(1) = *μ*(1). Therefore, we have *μ*(*x*∧(*x* → *y*)→(*x*⊙*y*)) = *μ*(1).

(4)⇒(1) From *x*⊙(*x* → *y*) ≤ *x*∧(*x* → *y*), it follows that *x*⊙  (*x* → *y*)→(*x*⊙  *y*) ≥ *x*∧(*x* → *y*)→(*x* ⊙  *y*). Then, we have *μ*(*x* ⊙ (*x* → *y*)→(*x* ⊙ *y*)) ≥ *μ*(*x* ∧(*x* → *y*)→(*x*⊙*y*)) = *μ*(1), which implies that *μ*(*x*⊙(*x* → *y*)→(*x* ⊙ *y*)) = *μ*(1). Taking *x* = *y*, we have *μ*(*x* → (*x*⊙*x*)) = *μ*(1). Therefore, *μ* is a fuzzy positive implicative filter by (2).

**Theorem 28**

Let *L* be a residuated *EQ*-algebra and *μ* be a fuzzy filter. The following are equivalent:

1. *μ* is a fuzzy positive implicative filter of *L*,
2. *μ*((*x*⊙*y*) → *z*) = *μ*((*x*∧*y*) → *z*) for all *x*, *y*, *z* ∈ *L*,
3. *μ*(*x*∧(*x* → *y*)→(*x*∧*y*)) = *μ*(1) for all *x*, *y* ∈ *L*,
4. *μ*(*x*∧(*x* → *y*)→(*y*∧(*y* → *x*))) = *μ*(1) for all *x*, *y* ∈ *L*.

**Proof**

  (1)⇔(2) Suppose that *μ* is a fuzzy positive implicative filter. We have *μ*(*y*∧(*y* → *z*) → *z*) = *μ*(1) by Theorem 26. Since *L* is a residuated *EQ*-algebra, then (*x*⊙*y*) → *z* ≤ *x* → (*y* → *z*) for all *x*, *y*, *z* ∈ *L* by Lemma 4 (15). So, *μ*((*x*⊙*y*) → *z*) ≤ *μ*(*x* → (*y* → *z*)). By Lemma 4 (10), we have *x* → (*y* → *z*)≤(*x*∧*y*)→(*y*∧(*y* → *z*)). Then, *μ*(*x* → (*y* → *z*)) ≤ *μ*((*x*∧*y*)→(*y*∧(*y* → *z*))). By Proposition 17, we get *μ*((*x*∧*y*) → *z*) ≥ *μ*((*x*∧*y*)→(*y*∧(*y* → *z*)))∧*μ*((*y*∧(*y* → *z*)) → *z*) ≥ *μ*((*x*⊙*y*) → *z*)∧*μ*(1) = *μ*((*x*⊙*y*) → *z*). On the other hand, since *x*⊙*y* ≤ *x*∧*y*, then (*x*∧*y*) → *z* ≤ (*x*⊙*y*) → *z*. It follows that *μ*((*x*∧*y*) → *z*) ≤ *μ*((*x*⊙*y*) → *z*). Consequently, we have *μ*((*x*⊙*y*) → *z*) = *μ*((*x*∧*y*) → *z*).

Conversely, since *L* is a residuated *EQ*-algebra, then it is a good *EQ*-algebra. It follows from Lemma 5 (3) that we have *x*⊙(*x* → *y*) ≤ *y*. Then, (*x*⊙(*x* → *y*)) → *y* = 1. So, *μ*((*x*⊙(*x* → *y*)) → *y*) = *μ*(1). Combining (2), we obtain *μ*((*x*⊙(*x* → *y*)) → *y*) = *μ*(*x*∧(*x* → *y*) → *y*) = *μ*(1). By Theorem 26, we have *μ* is a fuzzy positive implicative filter of *L*.

(1)⇒(3) Suppose that *μ* is a fuzzy positive implicative filter. Then, we have *μ*(*x*∧(*x* → *y*)→(*x* ⊙*y*)) = *μ*(1) for all *x*, *y* ∈ *L* by Theorem 27 (4). From *y* ≤ *x* → *y*, it follows that *x* ⊙*y* ≤ *x* ⊙(*x* → *y*). Then, we have *x*∧(*x* → *y*)→(*x* ⊙*y*) ≤ *x*∧(*x* → *y*) → *x*⊙(*x* → *y*). Hence, we get *μ*(1) = *μ*(*x*∧(*x* → *y*)→(*x*⊙*y*)) ≤ *μ*(*x*∧(*x* → *y*) → *x*⊙(*x* → *y*)), which implies that *μ*(*x*∧(*x* → *y*) → *x*⊙(*x* → *y*)) = *μ*(1). Moreover, by Lemma 5, we have *x*⊙(*x* → *y*) ≤ *x*∧*y*. Then, *x*∧(*x* → *y*)→(*x* ⊙(*x* → *y*)) ≤ *x* ∧(*x* → *y*)→(*x*∧*y*). It follows that *μ*(*x* ∧(*x* → *y*)→(*x* ⊙(*x* → *y*))) ≤ *μ*(*x* ∧(*x* → *y*)→(*x*∧*y*)). Therefore, *μ*(*x*∧(*x* → *y*)→(*x*∧*y*)) = *μ*(1).

(3)⇒(4) From *x* ≤ *y* → *x*, it follows that *x*∧*y* ≤ (*y* → *x*)∧*y*. Then, *x*∧(*x* → *y*)→(*x*∧*y*) ≤ *x*∧(*x* → *y*)→(*y*∧(*y* → *x*)). Hence, we have *μ*(*x*∧(*x* → *y*)→(*x*∧*y*)) ≤ *μ*(*x*∧(*x* → *y*)→(*y*∧(*y* → *x*))). Combining (3), we get *μ*(*x*∧(*x* → *y*)→(*y*∧(*y* → *x*))) = *μ*(1).

(4)⇒(1) By (4), we have *μ*(*x*∧(*x* → (*x*⊙*y*))→((*x*⊙*y*)∧((*x*⊙*y*) → *x*))) = *μ*(1). Then, *μ*(*x*∧(*x* → (*x*⊙*y*))→(*x*⊙*y*)) = *μ*(1). Since *L* is a residuated *EQ*-algebra, we get *y* ≤ *x* → (*x*⊙*y*) by Lemma 6 (3). It follows that *x*∧(*x* → (*x*⊙*y*))→(*x*⊙*y*) ≤ *x*∧*y* → (*x*⊙*y*). Then, *μ*(1) = *μ*(*x* ∧(*x* → (*x* ⊙*y*))→(*x* ⊙*y*)) ≤ *μ*(*x* ∧*y* → (*x* ⊙  *y*)), which implies that *μ*(*x*∧*y* → (*x*⊙*y*)) = *μ*(1). Therefore, *μ* is a fuzzy positive implicative filter of *L* by Theorem 27 (3).

As an application of Theorems 27 and 28, we can obtain some new characterizations of classical positive implicative filters in residuated *EQ*-algebras.

**Corollary 29**

Let *L* be a residuated *EQ*-algebra and let *F* be a filter of *L*. The following are equivalent:

1. *F* is a positive implicative filter of *L*,
2. *x* → (*x*⊙*x*) ∈ *F* for all *x* ∈ *L*,
3. *x*∧*y* → (*x*⊙*y*) ∈ *F* for all *x*, *y* ∈ *L*,
4. *x*∧(*x* → *y*)→(*x*⊙*y*) ∈ *F* for all *x*, *y* ∈ *L*,
5. *x*∧(*x* → *y*) → *y* ∈ *F* for all *x*, *y* ∈ *L*,
6. *x*∧(*x* → *y*)→(*x*∧*y*) ∈ *F* for all *x*, *y* ∈ *L*,
7. *x*∧(*x* → *y*)→(*y*∧(*y* → *x*)) ∈ *F* for all *x*, *y* ∈ *L*,
8. *x*∧(*x* → *y*) → *y* ∈ *F* for all *x*, *y* ∈ *L*.

The extension property for fuzzy positive implicative prefilters is obtained from the following proposition.

**Proposition 30**

Let *L* be a good *EQ*-algebra and let *μ* and *ν* be two fuzzy prefilters which satisfy *μ*⊆*ν*, *μ*(1) = *ν*(1). If *μ* is a fuzzy positive implicative prefilter, then so is *ν*.

**Proof**

We set *t* = *x* → (*x* → *y*); then, *x* → (*x* → (*t* → *y*)) = *t* → (*x* → (*x* → *y*)) = 1. Since *μ* is a fuzzy positive implicative prefilter, we have *μ*(*x* → (*t* → *y*)) = *μ*(*x* → (*x* → (*t* → *y*))) = *μ*(1) by Theorem 23. Then, *μ*(*x* → (*t* → *y*)) = *μ*(1) = *ν*(1). From *μ*⊆*ν*, it follows that *ν*(*x* → (*t* → *y*)) ≥ *μ*(*x* → (*t* → *y*)) = *ν*(1). So, *ν*(*x* → (*t* → *y*)) = *ν*(1). Since *ν* is a fuzzy prefilter, then *ν*(*x* → *y*) ≥ *ν*(*t* → (*x* → *y*))∧*ν*(*t*). It follows that *ν*(*x* → *y*) ≥ *ν*(1)∧*ν*(*t*) = *ν*(*t*) = *ν*(*x* → (*x* → *y*)). By Theorem 23, we get that *ν* is a fuzzy positive implicative prefilter.

**Proposition 31**

Let *L* be an *EQ*-algebra and let *μ* and *ν* be two fuzzy filters which satisfy *μ*⊆*ν*, *μ*(1) = *ν*(1). If *μ* is a fuzzy positive implicative filter, then so is *ν*.

**Proof**

It follows from Theorem 26.

## 5. Fuzzy Implicative Prefilters (Filters)

In this section, we introduce the notion of fuzzy implicative prefilters (filters) in *EQ*-algebras and discuss some of their properties.

**Definition 32**

Let *μ* be a fuzzy set in *L*. *μ* is called a* fuzzy implicative prefilter* of *L* if it satisfies

(FF1) *μ*(1) ≥ *μ*(*x*) for all *x* ∈ *L*,
(FF5) *μ*(*x*) ≥ *μ*(*z* → ((*x* → *y*) → *x*))∧*μ*(*z*) for all *x*, *y*, *z* ∈ *L*.

A fuzzy implicative prefilter *μ* of *L* is called a* fuzzy implicative filter* if it satisfies (FF3).

The following example shows that fuzzy implicative prefilters in *EQ*-algebras exist.

**Example 33**

Let *L* = {0, *a*, *b*, *c*, 1} be a chain with Cayley tables as Table 3.

Then, (*L*, ∧, ⊙, ~, 1) is an *EQ*-algebra in [27]. Define a fuzzy set *μ* in *L* as follows: *μ*(1) = *μ*(*a*) = *μ*(*b*) = *μ*(*c*) = *m* and *μ*(0) = *n*, where 0 ≤ *n* < *m* ≤ 1. One can check that *μ* is a fuzzy implicative prefilter of *L*.

**Lemma 34**

Let *μ* be a fuzzy implicative prefilter of *L*. For all *x*, *y* ∈ *L*, if *x* ≤ *y*, then *μ*(*x*) ≤ *μ*(*y*); that is, *μ* is order-preserving.

**Proof**

Let *μ* be a fuzzy implicative prefilter of *L*. By Definition 32, we have *μ*(*y*) ≥ *μ*(*x* → ((*y* → *y*) → *y*))∧*μ*(*x*). From (*y* → *y*) → *y* = 1 → *y* ≥ *y*, it follows that *x* → ((*y* → *y*) → *y*) ≥ *x* → *y*. If *x* ≤ *y*, then *x* → *y* = 1, which implies that *x* → ((*y* → *y*) → *y*) = 1. Therefore, we get *μ*(*y*) ≥ *μ*(1)∧*μ*(*x*) = *μ*(*x*); that is, *μ* is order-preserving.

The following result describes the relationship between fuzzy implicative prefilters (filters) and fuzzy prefilters (filters).

**Theorem 35**

Each fuzzy implicative prefilter of *L* is a fuzzy prefilter.

**Proof**

Suppose that *μ* is a  fuzzy implicative prefilter of *L*. Then, *μ*(1) ≥ *μ*(*x*) for all *x* ∈ *L*. From *y* ≤ 1 → *y*, we get that *x* → *y* ≤ *x* → (1 → *y*). By Lemma 34, we have *μ*(*x* → *y*) ≤ *μ*(*x* → (1 → *y*)). It follows from Definition 32 that *μ*(*y*) ≥ *μ*(*x* → ((*y* → 1) → *y*))∧*μ*(*x*) = *μ*(*x* → (1 → *y*))∧*μ*(*x*) ≥ *μ*(*x* → *y*)∧*μ*(*x*), which implies that (FF2) holds. Therefore, *μ* is a fuzzy prefilter of *L*.

In the following example, we show that the converse of above theorem is not correct.

**Example 36**

Let *L* be the *EQ*-algebra defined in Example 12. Define a fuzzy set *μ* in *L* as follows: *μ*(1) = 0.9 and *μ*(0) = *μ*(*a*) = *μ*(*b*) = 0.7. One can check that *μ* is a fuzzy prefilter of *L*. However, since *μ*(*a*) < *μ*(1)∧*μ*(1 → ((*a* → 0) → *a*)), then *μ* is not a fuzzy implicative prefilter of *L*.

Our next aim is to show some characterizations of fuzzy prefilters (filters).

**Theorem 37**

Let *μ* be a fuzzy prefilter (filter) in *L*. The following are equivalent:

1. *μ* is a fuzzy implicative prefilter (filter) of *L*,
2. *μ*(*x*) ≥ *μ*((*x* → *y*) → *x*) for all *x*, *y* ∈ *L*,
3. *μ*(*x*) = *μ*((*x* → *y*) → *x*) for all *x*, *y* ∈ *L*.

**Proof**

  (1)⇒(2) Suppose  that *μ* is a fuzzy implicative prefilter of *L*. Then, *μ*(1) ≥ *μ*(*x*) for all *x* ∈ *L*. From Definition 32, we have *μ*(*x*) ≥ *μ*(1 → ((*x* → *y*) → *x*))∧*μ*(1) = *μ*(1 → ((*x* → *y*) → *x*)). Since (*x* → *y*) → *x* ≤ 1 → ((*x* → *y*) → *x*)), then *μ*(1 → ((*x* → *y*) → *x*)) ≥ *μ*((*x* → *y*) → *x*). Consequently, we have *μ*(*x*) ≥ *μ*(1 → ((*x* → *y*) → *x*)) ≥ *μ*((*x* → *y*) → *x*).

(2)⇒(3) From *x* ≤ (*x* → *y*) → *x*, it follows that *μ*(*x*) ≤ *μ*((*x* → *y*) → *x*). Combining (2), we get *μ*(*x*) = *μ*((*x* → *y*) → *x*).

(3)⇒(1) Let *μ* be a fuzzy prefilter in *L*. Then, *μ*(1) ≥ *μ*(*x*) for all *x* ∈ *L*. By Definition 10, we have *μ*((*x* → *y*) → *x*) ≥ *μ*(*z*)∧*μ*(*z* → ((*x* → *y*) → *x*)). Combining (3), we obtain *μ*(*x*) ≥ *μ*(*z*)∧*μ*(*z* → ((*x* → *y*) → *x*)). By Definition 32, we have *μ* is a fuzzy implicative prefilter of *L*.

As a consequence of Theorem 37, we have the following corollary.

**Corollary 38**

Let *L* be an *EQ*-algebra with a bottom element 0 and let *μ* be a fuzzy prefilter (filter) in *L*. The following are equivalent:

1. *μ* is a fuzzy implicative prefilter (filter) of *L*,
2. *μ*(*x*) ≥ *μ*(¬*x* → *x*) for all *x* ∈ *L*,
3. *μ*(*x*) = *μ*(¬*x* → *x*) for all *x* ∈ *L*.

**Proof**

  (1)⇒(2)  Assume that *μ* is a fuzzy implicative prefilter of *L*. Then, *μ*(*x*) ≥ *μ*((*x* → 0) → *x*) = *μ*(¬*x* → *x*) by Theorem 37.

(2)⇒(3) Since *x* ≤ ¬*x* → *x*, then *μ*(*x*) ≤ *μ*(¬*x* → *x*) as *μ* is a fuzzy prefilter in *L*. Combining *μ*(*x*) ≥ *μ*(¬*x* → *x*), we get *μ*(*x*) = *μ*(¬*x* → *x*).

(3)⇒(1) From *x* → 0 ≤ *x* → *y*, it follows that (*x* → *y*) → *x* ≤ ¬*x* → *x*. Then, *μ*((*x* → *y*) → *x*) ≤ *μ*(¬*x* → *x*) as *μ* is a fuzzy prefilter. Combining (3), we get *μ*(*x*) ≥ *μ*((*x* → *y*) → *x*). Therefore, we have that *μ* is a fuzzy implicative prefilter of *L* by Theorem 37.

**Theorem 39**

Let *μ* be a fuzzy set in *L*. *μ* is a fuzzy implicative prefilter (filter) of *L* if and only if, for all *t* ∈ [0,1], *μ*_*t*_ is either empty or an implicative prefilter (filter) of *L*.

**Proof**

The proof is easy, and we hence omit the details.

**Corollary 40**

Let *F* be a nonempty subset of *L*. *F* is an implicative prefilter (filter) if and only if *χ*_*F*_ is a fuzzy implicative prefilter (filter).

**Definition 41**

Let *μ* be a fuzzy prefilter in *L*. We call that *μ* has* weak exchange principle* if it satisfies for all *x*, *y*, *z* ∈ *L* *μ*(*x* → (*y* → *z*)) = *μ*(*y* → (*x* → *z*)).

**Example 42**

From Lemma 5, we know that any fuzzy prefilter *μ* of a good *EQ*-algebra *L* has weak exchange principle.

**Example 43**

Let *L* be the *EQ*-algebra and *μ* be the fuzzy set of *L* defined in Example 12. One can check that *L* is not a good *EQ*-algebra and *μ* is a fuzzy prefilter of *L*, which has weak exchange principle.

We have the following characterizations of fuzzy implicative prefilters in an *EQ*-algebra with a bottom element 0.

**Theorem 44**

Let *L* be an *EQ*-algebra with a bottom element 0 and let *μ* be a fuzzy prefilter with weak exchange principle. The following are equivalent:

1. *μ* is a fuzzy implicative prefilter of *L*,
2. *μ*(*x* → *z*) ≥ *μ*(*x* → (¬*z* → *y*))∧*μ*(*y* → *z*) for all *x*, *y*, *z* ∈ *L*,
3. *μ*(*x* → *z*) ≥ *μ*(*x* → (¬*z* → *z*)) for all *x*, *z* ∈ *L*,
4. *μ*(*x* → *z*) = *μ*(*x* → (¬*z* → *z*)) for all *x*, *z* ∈ *L*.

**Proof**

  (1)⇔(2) Since *y* → *z* ≤ (*x* → *y*)→(*x* → *z*) and ¬*z* → (*x* → *y*)≤((*x* → *y*)→(*x* → *z*))→(¬*z* → (*x* → *z*)), then *μ*(*y* → *z*) ≤ *μ*((*x* → *y*)→(*x* → *z*)) and *μ*(¬*z* → (*x* → *y*)) ≤ *μ*(((*x* → *y*)→(*x* → *z*))→(¬*z* → (*x* → *z*))). From Definition 10, it follows that *μ*(¬*z* → (*x* → *z*)) ≥ *μ*((*x* → *y*)→(*x* → *z*))∧*μ*(((*x* → *y*)→(*x* → *z*))→(¬*z* → (*x* → *z*))) ≥ *μ*(*y* → *z*)∧*μ*(¬*z* → (*x* → *y*)) ≥ *μ*(*y* → *z*)∧*μ*(*x* → (¬*z* → *y*)). Notice that ¬(*x* → *z*)≤¬*z*, and we have ¬*z* → (*x* → *z*)≤¬(*x* → *z*)→(*x* → *z*). Then, *μ*(¬*z* → (*x* → *z*)) ≤ *μ*(¬(*x* → *z*)→(*x* → *z*)). Since *μ* is a fuzzy implicative prefilter of *L*, we have *μ*(*x* → *z*) ≥ *μ*(¬(*x* → *z*)→(*x* → *z*)) by Corollary 38. Consequently, we obtain *μ*(*x* → *z*) ≥ *μ*(*x* → (¬*z* → *y*))∧*μ*(*y* → *z*).

Conversely, suppose that *μ* satisfies *μ*(*x* → *z*) ≥ *μ*(*x* → (¬*z* → *y*))∧*μ*(*y* → *z*) for all *x*, *y*, *z* ∈ *L*. Then, *μ*(1 → *x*) ≥ *μ*(1 → (¬*x* → *x*))∧*μ*(*x* → *x*) ≥ *μ*(¬*x* → *x*)∧*μ*(1) = *μ*(¬*x* → *x*). Since *μ* is a fuzzy prefilter, then *μ*(*x*) ≥ *μ*(1)∧*μ*(1 → *x*) = *μ*(1 → *x*). It follows that *μ*(*x*) ≥ *μ*(¬*x* → *x*). By Corollary 38, we get that *μ* is a fuzzy implicative prefilter of *L*.

(1)⇒(3) Suppose that *μ* is a fuzzy implicative prefilter of *L*. Then, we have *μ*(*x* → *z*) ≥ *μ*(*x* → (¬*z* → *z*))∧*μ*(*z* → *z*) = *μ*(*x* → (¬*z* → *z*))∧*μ*(1) by (2). Therefore, we obtain *μ*(*x* → *z*) ≥ *μ*(*x* → (¬*z* → *z*).

(3)⇒(4) From *z* ≤ ¬*z* → *z*, it follows that *x* → *z* ≤ *x* → (¬*z* → *z*). Then, *μ*(*x* → *z*) ≤ *μ*(*x* → (¬*z* → *z*)) as *μ* is a fuzzy prefilter. Combining (3), we get *μ*(*x* → *z*) = *μ*(*x* → (¬*z* → *z*)).

(4)⇒(1) Let *μ* be a fuzzy prefilter which satisfies *μ*(*x* → *z*) = *μ*(*x* → (¬*z* → *z*)) for all *x*, *z* ∈ *L*. Then, *μ*(1 → *x*) = *μ*(1 → (¬*x* → *x*)). Since *μ* is a fuzzy prefilter, then *μ*(*x*) ≥ *μ*(1 → *x*)∧*μ*(1) = *μ*(1 → *x*). From ¬*x* → *x* ≤ 1 → (¬*x* → *x*), it follows that *μ*(1 → (¬*x* → *x*)) ≥ *μ*(¬*x* → *x*). Consequently, we obtain *μ*(*x*) ≥ *μ*(¬*x* → *x*). Using Corollary 38, we get that *μ* is a fuzzy implicative prefilter of *L*.

## 6. The Relations among Special Fuzzy Prefilters (Filters)

In this section, we introduce fuzzy fantastic prefilters (filters) in *EQ*-algebras and discuss the relations among various fuzzy prefilters (filters). Particularly, we find some conditions under which a fuzzy implicative prefilter (filter) is equivalent to a fuzzy positive implicative prefilter (filter). Moreover, as applications, we obtain some relations among corresponding classical filters in *EQ*-algebras and some related results about fuzzy filters in residuated lattices.

**Definition 45**

Let *F* be a prefilter in *L*. *F* is called a* fantastic prefilter* of *L* if it satisfies the following:

(F6) *y* → *x* ∈ *F* implies that ((*x* → *y*) → *y*) → *x* ∈ *F* for all *x*, *y* ∈ *L*.

If *F* is a filter and it satisfies (F6), then it is called a* fantastic filter* of *L*.

It is time to give some examples of fantastic prefilters.

**Example 46**

Let *L* = {0, *a*, *b*, 1} be a chain with Cayley tables as Table 4.

Then, (*L*, ∧, ⊙, ~, 1) is an *EQ*-algebra. One can check that *F* = {1} is a fantastic prefilter.

**Definition 47**

Let *μ* be a fuzzy prefilter in *L*. *μ* is called a* fuzzy fantastic prefilter* of *L* if it satisfies

(FF6) *μ*(((*x* → *y*) → *y*) → *x*) ≥ *μ*(*y* → *x*) for all *x*, *y* ∈ *L*.

A fuzzy filter *μ* of *L* is called a* fuzzy fantastic filter* if it satisfies (FF6).

**Example 48**

Let (*L*, ∧, ⊙, ~, 1) be an *EQ*-algebra in Example 46. Define a fuzzy set *μ* in *L* as follows: *μ*(1) = 0.7 and *μ*(0) = *μ*(*a*) = *μ*(*b*) = 0.4. One can check that *μ* is a fuzzy fantastic prefilter of *L*.

Next, we derive a characterization of fuzzy fantastic prefilters (filters).

**Theorem 49**

Let *μ* be a fuzzy prefilter (filter) in *L*. Then, *μ* is a fuzzy fantastic prefilter (filter) of *L* if and only if *μ*(((*x* → *y*) → *y*) → *x*) ≥ *μ*(*z* → (*y* → *x*))  ∧  *μ*(*z*) for all *x*, *y*, *z* ∈ *L*.

**Proof**

Suppose that *μ* is  a fuzzy fantastic prefilter of *L*. From Definition 45, it follows that *μ*(((*x* → *y*) → *y*) → *x*) ≥ *μ*(*y* → *x*) for all *x*, *y* ∈ *L*. Since *μ* is a fuzzy prefilter in *L*, then *μ*(*y* → *x*) ≥ *μ*(*z* → (*y* → *x*))∧*μ*(*z*). Therefore, we obtain *μ*(((*x* → *y*) → *y*) → *x*) ≥ *μ*(*z* → (*y* → *x*))∧*μ*(*z*).

Conversely, suppose that *μ* satisfies *μ*(((*x* → *y*) → *y*) → *x*) ≥ *μ*(*z* → (*y* → *x*))∧*μ*(*z*) for all *x*, *y*, *z* ∈ *L*. Taking *z* = 1, we obtain *μ*(((*x* → *y*) → *y*) → *x*)) ≥ *μ*(1 → (*y* → *x*))∧*μ*(1) = *μ*(1 → (*y* → *x*)). From *y* → *x* ≤ 1 → (*y* → *x*), it follows that *μ*(*y* → *x*) ≤ *μ*(1 → (*y* → *x*)). Consequently, we obtain *μ*(((*x* → *y*) → *y*) → *x*) ≥ *μ*(*y* → *x*). Therefore, *μ* is a fuzzy fantastic prefilter of *L*.

**Theorem 50**

Let *μ* be a fuzzy set in *L*. *μ* is a fuzzy fantastic prefilter (filter) of *L* if and only if, for all *t* ∈ [0,1], *μ*_*t*_ is either empty or a fantastic prefilter (filter) of *L*.

**Proof**

The proof is easy, and we hence omit the details.

**Corollary 51**

Let *F* be a nonempty subset of *L*. *F* is a fantastic prefilter (filter) if and only if *χ*_*F*_ is a fuzzy fantastic prefilter (filter).

In what follows, we pay attention to the relations among various special fuzzy prefilters (filters).

First, the relationship between fuzzy implicative prefilters and fuzzy fantastic prefilters can be described by the following theorem.

**Theorem 52**

Each fuzzy implicative prefilter (filter) with weak exchange principle is a fuzzy fantastic prefilter (filter) in an *EQ*-algebra.

**Proof**

Suppose that *μ*  is a fuzzy implicative prefilter with weak exchange principle. From *x* ≤ ((*x* → *y*) → *y*) → *x*, it follows that (((*x* → *y*) → *y*) → *x*) → *y* ≤ *x* → *y*. This implies that ((((*x* → *y*) → *y*) → *x*) → *y*)→(((*x* → *y*) → *y*) → *x*)≥(*x* → *y*)→(((*x* → *y*) → *y*) → *x*). Since *μ* is a fuzzy implicative prefilter, then *μ*(((((*x* → *y*) → *y*) → *x*) → *y*)→(((*x* → *y*) → *y*) → *x*)) ≥ *μ*((*x* → *y*)→(((*x* → *y*) → *y*) → *x*)) by Lemma 34. We can obtain that *μ*((*x* → *y*)→(((*x* → *y*) → *y*) → *x*)) = *μ*(((*x* → *y*) → *y*)→((*x* → *y*) → *x*)) as *μ* has weak exchange principle. From *y* → *x* ≤ ((*x* → *y*) → *y*)→((*x* → *y*) → *x*), it follows that *μ*(*y* → *x*) ≤ *μ*(((*x* → *y*) → *y*)→((*x* → *y*) → *x*)). Together with them, we obtain *μ*(((((*x* → *y*) → *y*) → *x*) → *y*)→(((*x* → *y*) → *y*) → *x*)) ≥ *μ*(*y* → *x*). On the other hand, since *μ* is a fuzzy implicative prefilter, then *μ*(((*x* → *y*) → *y*) → *x*) ≥ *μ*(1 → ((((*x* → *y*) → *y*) → *x*) → *y*)→(((*x* → *y*) → *y*) → *x*))∧*μ*(1) = *μ*(1 → ((((*x* → *y*) → *y*) → *x*) → *y*)→(((*x* → *y*) → *y*) → *x*)) ≥ *μ*(((((*x* → *y*) → *y*) → *x*) → *y*)→(((*x* → *y*) → *y*) → *x*)). Consequently, we obtain *μ*(((*x* → *y*) → *y*) → *x*) ≥ *μ*(*y* → *x*). Therefore, we get that *μ* is a fuzzy fantastic prefilter by Definition 45.

From the following example, we can see that the converse of Theorem 52 may not be true.

**Example 53**

Let *L* be the *EQ*-algebra and let *μ* be the fuzzy set of *L* defined in Example 48. We know that *μ* is a fuzzy fantastic prefilter of *L*. But it is not a fuzzy implicative prefilter of *L*, since *μ*(*b*) < *μ*((*b* → 0) → *b*).

Next, the following theorems show the relationship between fuzzy implicative prefilters (filters) and fuzzy positive implicative prefilters (filters).

**Theorem 54**

Each fuzzy implicative prefilter with weak exchange principle is a fuzzy positive implicative prefilter in an *EQ*-algebra.

**Proof**

Suppose that  *μ* is a fuzzy implicative prefilter of *L*. Then, *μ* is a fuzzy prefilter of *L* by Theorem 35. It follows that *μ*((*x* → (*x* → *z*)) ≥ *μ*(*x* → *y*)∧*μ*((*x* → *y*)→(*x* → (*x* → *z*))). Since *y* → (*x* → *z*)≤(*x* → *y*)→(*x* → (*x* → *z*)), then *μ*(*y* → (*x* → *z*)) ≤ *μ*((*x* → *y*)→(*x* → (*x* → *z*))). In a similar way, we get that *μ*(*x* → (*x* → *z*)) ≤ *μ*(((*x* → *x*) → *z*)→(*x* → *z*)). Hence, we obtain *μ*(((*x* → *x*) → *z*)→(*x* → *z*)) ≥ *μ*(*x* → *y*)∧*μ*(*y* → (*x* → *z*)) = *μ*(*x* → *y*)∧*μ*(*x* → (*y* → *z*)) as *μ* has weak exchange principle. From ((*x* → *z*) → *z*)→(*x* → *z*) ≤ 1 → (((*x* → *z*) → *z*)→(*x* → *z*)), it follows that *μ*(((*x* → *z*) → *z*)→(*x* → *z*)) ≤ *μ*(1 → (((*x* → *z*) → *z*)→(*x* → *z*))). Since *μ* is a fuzzy implicative prefilter of *L*, then *μ*(*x* → *z*) ≥ *μ*(1 → (((*x* → *z*) → *z*)→(*x* → *z*)))∧*μ*(1) = *μ*(1 → (((*x* → *z*) → *z*)→(*x* → *z*))). Consequently, we obtain *μ*(*x* → *z*) ≥ *μ*(*x* → *y*)∧*μ*(*x* → (*y* → *z*)). Therefore, *μ* is a fuzzy positive implicative prefilter.

**Theorem 55**

Each fuzzy implicative filter is a fuzzy positive implicative filter in an *EQ*-algebra.

**Proof**

From *x*∧(*x* → *y*) ≤ *x* and *x*∧(*x* → *y*) ≤ *x* → *y*, it follows that *x*∧(*x* → *y*) ≤ *x* → *y* ≤ (*x*∧(*x* → *y*)) → *y*. Then, we have ((*x*∧(*x* → *y*)) → *y*) → *y* ≤ (*x*∧(*x* → *y*)) → *y*, which implies that ((*x*∧(*x* → *y*)) → *y*) → *y*)→((*x*∧(*x* → *y*)) → *y*) = 1. Hence, we obtain *μ*(((*x*∧(*x* → *y*)) → *y*) → *y*)→((*x*∧(*x* → *y*)) → *y*)) = *μ*(1). Since *μ* is a fuzzy implicative filter of *L*, then *μ*(((*x*∧(*x* → *y*)) → *y*) → *y*)→((*x*∧(*x* → *y*)) → *y*)) = *μ*((*x*∧(*x* → *y*)) → *y*) by Theorem 37. It follows that *μ*((*x*∧(*x* → *y*)) → *y*) = *μ*(1). Therefore, we get that *μ* is a fuzzy positive implicative filter by Theorem 26.

The following example shows that the converse of the above theorem may not be true.

**Example 56**

Let *L* be the *EQ*-algebra and let *μ* be the fuzzy set of *L* defined in Example 19. We know that *μ* is a fuzzy positive implicative filter of *L*. But it is not a fuzzy implicative filter of *L*, since *μ*(*a*) < *μ*((*a* → 0) → *a*).

The following result displays the relations among fuzzy positive implicative prefilters (filters), fuzzy implicative prefilters (filters), and fuzzy fantastic prefilters (filters). Also, it provides a condition under which the converse of Theorems 54 and 55 can be true.

**Theorem 57**

Let *L* be a good *EQ*-algebra. Then, *μ* is a fuzzy implicative prefilter (filter) if and only if *μ* is both a fuzzy positive implicative prefilter (filter) and a fuzzy fantastic prefilter (filter) of *L*.

**Proof**

Suppose  that *μ* is a fuzzy implicative prefilter. Since *L* is a good *EQ*-algebra, then *μ* has weak exchange principle. Hence, we have that *μ* is a fuzzy fantastic prefilter of *L* by Theorem 54. Moreover, using Theorem 55, we get that *μ* is a fuzzy positive implicative prefilter.

Conversely, suppose that *μ* is both a fuzzy positive implicative prefilter and a fuzzy fantastic prefilter of *L*. Since *μ* is a fuzzy positive implicative prefilter, then *μ*((*x* → *y*) → *y*) ≥ *μ*((*x* → *y*)→((*x* → *y*) → *y*)) by Theorem 23. Notice that *L* is a good *EQ*-algebra; we obtain *x* ≤ (*x* → *y*) → *y* by Lemma 5 (2). Then, (*x* → *y*) → *x* ≤ (*x* → *y*)→((*x* → *y*) → *y*). It follows that *μ*((*x* → *y*) → *x*) ≤ *μ*((*x* → *y*)→((*x* → *y*) → *y*)). Consequently, we obtain *μ*((*x* → *y*) → *y*) ≥ *μ*((*x* → *y*) → *x*). On the other hand, since *μ* is a fuzzy fantastic prefilter of *L*, we get *μ*(((*x* → *y*) → *y*) → *x*)) ≥ *μ*(*y* → *x*) by Definition 47. From (*x* → *y*) → *x* ≤ *y* → *x*, it follows that *μ*(*y* → *x*) ≥ *μ*((*x* → *y*) → *x*), which implies that *μ*(((*x* → *y*) → *y*) → *x*) ≥ *μ*((*x* → *y*) → *x*). Moreover, since *μ* is a fuzzy prefilter of *L*, we have *μ*(*x*) ≥ *μ*(((*x* → *y*) → *y*) → *x*)∧*μ*((*x* → *y*) → *y*) and *μ*((*x* → *y*) → *x*) ≥ *μ*(*z* → ((*x* → *y*) → *x*))∧*μ*(*z*). Consequently, we obtain *μ*(*x*) ≥ *μ*((*x* → *y*) → *x*)∧*μ*((*x* → *y*) → *x*) = *μ*((*x* → *y*) → *x*) ≥ *μ*((*z* → ((*x* → *y*) → *x*))∧*μ*(*z*). Therefore, *μ* is a fuzzy implicative prefilter of *L* by Definition 32.

Combining Corollaries 25, 40 and 51 and Theorem 57 and taking the special case of fuzzy prefilters (filters), we can obtain the relations among positive implicative prefilters (filters), implicative prefilters (filters), and fantastic prefilters (filters) as corollaries.

**Corollary 58**

Let *L* be a good *EQ*-algebra and let *F* be a nonempty subset of *L*. Then, *F* is an implicative prefilter (filter) if and only if *F* is both a positive implicative prefilter (filter) and a fantastic prefilter (filter) of *L*.

Since residuated *EQ*-algebras are good *EQ*-algebras, in view of Corollary 58, we have the following.

**Corollary 59**

Let *L* be a residuated *EQ*-algebra and let *F* be a fantastic filter of *L*. Then, *F* is an implicative filter if and only if *F* is a positive implicative filter of *L*.

**Corollary 60**

Let *L* be a residuated *EQ*-algebra and let *F* be a positive implicative filter of *L*. Then, *F* is an implicative filter if and only if *F* is a fantastic filter of *L*.

The above results sufficiently show that fuzzy filters are a useful tool to obtain results on classical filters. Moreover, the above results further develop the classical filter theory in *EQ*-algebras.

In what follows, we continue to find the conditions under which a fuzzy positive implicative prefilter (filter) is a fuzzy implicative prefilter (filter).

**Lemma 61**

Let *F* be a positive implicative prefilter of an *EQ*-algebra *L*. Then, for all *a* ∈ *L*, *F*_*a*_ = {*x* ∈ *L* | *a* → *x* ∈ *F*} is the least prefilter containing *F* and *a*.

**Proof**

Suppose that *F* is a positive implicative prefilter of *L*. Since *a* → 1 = 1 ∈ *F*, then 1 ∈ *F*_*a*_. For all *x*, *y* ∈ *L*, if *x*, *x* → *y* ∈ *F*_*a*_, then *a* → *x* ∈ *F* and *a* → (*x* → *y*) ∈ *F*. Since *F* is a positive implicative prefilter, we have *a* → *y* ∈ *F*; that is, *y* ∈ *F*_*a*_. Therefore, *F*_*a*_ is a prefilter in *L*.

For any *x* ∈ *L*, if *x* ∈ *F*, since *x* ≤ *a* → *x*, and *F* is a prefilter, we have *a* → *x* ∈ *F*; that is, *x* ∈ *F*_*a*_. Hence, *F*⊆*F*_*a*_. Moreover, since *a* → *a* = 1 ∈ *F*, then *a* ∈ *F*_*a*_. Therefore, *F* ∪ {*a*}⊆*F*_*a*_. Now, let *G* be a prefilter of *L* such that *F* ∪ {*a*}⊆*G*; then, for any *x* ∈ *F*_*a*_, we have *a* → *x* ∈ *F*⊆*G*. Since *a* ∈ *G* and *G* is a prefilter in *L*, we have *x* ∈ *G*; that is, *F*_*a*_⊆*G*. Therefore, *F*_*a*_ is the least prefilter containing *F* and *a*.

A prefilter *F* of an *EQ*-algebra *L* is called* maximal* if and only if it is proper and no proper prefilter of *L* strictly contains *F*; that is, for each prefilter, *G* ≠ *F*; if *F*⊆*G* then *G* = *L*.

**Theorem 62**

Let *μ* be a fuzzy prefilter with weak exchange principle in an *EQ*-algebra *L*. The following are equivalent:

1. *μ* is a fuzzy implicative prefilter and *μ*_*μ*(1)_ is a maximal prefilter in *L*,
2. *μ* is a fuzzy positive implicative prefilter and *μ*_*μ*(1)_ is a maximal prefilter in *L*,
3. *μ* satisfies that *μ*(*x*) ≠ *μ*(1) and *μ*(*y*) ≠ *μ*(1) can imply that *μ*(*x* → *y*) = *μ*(1) and *μ*(*y* → *x*) = *μ*(1) for all *x*, *y* ∈ *L*.

**Proof**

  (1)⇒(2) It follows from Theorem 54.

(2)⇒(3) Suppose that *μ*(*x*) ≠ *μ*(1) and *μ*(*y*) ≠ *μ*(1); thus, *x* ∉ *μ*_*μ*(1)_ and *y* ∉ *μ*_*μ*(1)_. Since *μ* is a fuzzy positive implicative prefilter of *L*, we can easily prove that *μ*_*μ*(1)_ is a positive implicative prefilter in *L*. It follows from Lemma 61 that *F*_*y*_ = {*t* ∈ *L* | *y* → *t* ∈ *μ*_*μ*(1)_} is the least prefilter containing *μ*_*μ*(1)_ and *y*. Notice that *μ*_*μ*(1)_ is a maximal prefilter in *L*; we get *F*_*y*_ = *L*. Hence, *x* ∈ *F*_*y*_; that is, *y* → *x* ∈ *μ*_*μ*(1)_, which implies that *μ*(*y* → *x*) = *μ*(1). In a similar way, we can get *μ*(*x* → *y*) = *μ*(1).

(3)⇒(1) Assume on the contrary that *μ* is not a fuzzy implicative prefilter of *L*. Then, by Theorem 37, there exist *x*, *y* ∈ *L* such that *μ*(*x*) < *μ*((*x* → *y*) → *x*). Hence, *μ*(*x*) ≠ *μ*(1). Now, we consider two cases: either *μ*(*y*) = *μ*(1) or *μ*(*y*) ≠ *μ*(1).

If *μ*(*y*) = *μ*(1), since *μ*(*y*) ≤ *μ*(*x* → *y*), then *μ*(*x* → *y*) = *μ*(1). Since *μ* is a fuzzy prefilter in *L*, then *μ*(*x*) ≥ *μ*((*x* → *y*) → *x*)∧*μ*(*x* → *y*) = *μ*((*x* → *y*) → *x*).

If *μ*(*y*) ≠ *μ*(1), combining *μ*(*x*) ≠ *μ*(1), we have *μ*(*x* → *y*) = *μ*(1) by assumption. Similarly, we have *μ*(*x*) ≥ *μ*((*x* → *y*) → *x*).

In any case, we have a contradiction. Therefore, *μ* is a fuzzy implicative prefilter of *L*. It follows from Theorem 54 that *μ* is a fuzzy positive implicative prefilter of *L*. Hence, *μ*_*μ*(1)_ is a positive implicative prefilter in *L*. By Lemma 61, we have that *F*_*a*_ = {*x* ∈ *L* | *a* → *x* ∈ *μ*_*μ*(1)_} is the least prefilter containing *μ*_*μ*(1)_ and *a*.

In order to prove that *μ*_*μ*(1)_ is a maximal prefilter in *L*, it is sufficient to show that, for all *a* ∈ *L* − *μ*_*μ*(1)_, *F*_*a*_ = {*x* ∈ *L* | *a* → *x* ∈ *μ*_*μ*(1)_} = *L*. For all *t* ∈ *L*, if *t* ∈ *μ*_*μ*(1)_, then *t* ∈ *F*_*a*_ = {*x* ∈ *L* | *a* → *x* ∈ *μ*_*μ*(1)_}. If *t* ∉ *μ*_*μ*(1)_, then *μ*(*t*) ≠ *μ*(1). Since *a* ∉ *μ*_*μ*(1)_, then *μ*(*a*) ≠ *μ*(1). It follows from (3) that *μ*(*a* → *t*) = *μ*(1); that is, *a* → *t* ∈ *μ*_*μ*(1)_, which implies that *t* ∈ *F*_*a*_ = {*x* ∈ *L* | *a* → *x* ∈ *μ*_*μ*(1)_}. In any case, we have *F*_*a*_ = *L*. Therefore, *μ*_*μ*(1)_ is a maximal prefilter in *L*. This completes the proof.

**Corollary 63**

Let *L* be a good *EQ*-algebra. Suppose that *μ* is a fuzzy prefilter and *μ*_*μ*(1)_ is a maximal prefilter in *L*. Then, *μ* is a fuzzy implicative prefilter of *L* if and only if it is a fuzzy positive implicative prefilter of *L*.

Next, we further find the conditions under which a fuzzy positive implicative filter is equivalent to a fuzzy implicative filter.

**Theorem 64**

Let *L* be a good *EQ*-algebra with a bottom element 0 and let *μ* be a fuzzy positive implicative filter. Then, *μ* is a fuzzy implicative filter if and only if it satisfies *μ*(¬¬*x*) = *μ*(*x*) for all *x* ∈ *L*.

**Proof**

Suppose that *μ* is a fuzzy implicative filter. From ¬¬*x* = ¬*x* → 0 ≤ ¬*x* → *x*, it follows that *μ*(¬*x* → *x*) ≥ *μ*(¬¬*x*). By Corollary 38, we have *μ*(*x*) = *μ*(¬*x* → *x*). Consequently, we obtain *μ*(*x*) ≥ *μ*(¬¬*x*). Since *L* is a good *EQ*-algebra with a bottom element 0, then *x* ≤ (*x* → 0) → 0 by Lemma 5; that is, *x* ≤ ¬¬*x*. It follows that *μ*(*x*) ≤ *μ*(¬¬*x*). Therefore, we get *μ*(¬¬*x*) = *μ*(*x*).

Conversely, suppose that *μ* is a fuzzy positive implicative filter and satisfies *μ*(¬¬*x*) = *μ*(*x*) for all *x* ∈ *L*. Since ¬*x* → *x* ≤ (*x* → 0)→(¬*x* → 0) = ¬*x* → (¬*x* → 0), then *μ*(¬*x* → (¬*x* → 0)) ≥ *μ*(¬*x* → *x*). It follows from Theorem 23 that *μ*(¬*x* → 0) = *μ*(¬*x* → (¬*x* → 0)). So we have *μ*(¬¬*x*) ≥ *μ*(¬*x* → *x*). Combining *μ*(¬¬*x*) = *μ*(*x*), we obtain *μ*(*x*) ≥ *μ*(¬*x* → *x*). Therefore, *μ* is a fuzzy implicative filter of *L* by Corollary 38.

Notice that an involutive *EQ*-algebra *L* satisfies ¬¬*x* = *x* for all *x* ∈ *L*. We have the following.

**Corollary 65**

Let *L* be a good involutive *EQ*-algebra. Then, *μ* is a fuzzy implicative filter of *L* if and only if it is a fuzzy positive implicative filter of *L*.

Since involutive residuated lattices are involutive residuated *EQ*-algebras, which are good involutive *EQ*-algebras, we can obtain some related results about fuzzy filters in residuated lattices.

**Corollary 66**

Let *L* be an involutive residuated lattice and let *μ* be a fuzzy filter of *L*. Then, the following are equivalent:

1. *μ* is a fuzzy implicative filter of *L*,
2. *μ* is a fuzzy positive implicative filter of *L*,
3. *μ* ^*a*^ is a fuzzy filter for all *a* ∈ *L*,
4. *μ*(*x* → *y*) ≥ *μ*(*x* → (*x* → *y*)) for all *x*, *y* ∈ *L*,
5. *μ*(*x* → *y*) = *μ*(*x* → (*x* → *y*)) for all *x*, *y* ∈ *L*,
6. *μ*(*x*∧(*x* → *y*) → *y*) = *μ*(1) for all *x*, *y* ∈ *L*,
7. *μ*(*x* → (*x*⊙*x*)) = *μ*(1) for all *x* ∈ *L*,
8. *μ*(*x*∧*y* → (*x*⊙*y*)) = *μ*(1) for all *x*, *y* ∈ *L*,
9. *μ*(*x*∧(*x* → *y*)→(*x*⊙*y*)) = *μ*(1) for all *x*, *y* ∈ *L*,
10. *μ*((*x*⊙*y*) → *z*) = *μ*((*x*∧*y*) → *z*) for all *x*, *y*, *z* ∈ *L*,
11. *μ*(*x*∧(*x* → *y*)→(*x*∧*y*)) = *μ*(1) for all *x*, *y* ∈ *L*,
12. *μ*(*x*∧(*x* → *y*)→(*y*∧(*y* → *x*))) = *μ*(1) for all *x*, *y* ∈ *L*,
13. *μ*(*x*) ≥ *μ*((*x* → *y*) → *x*) for all *x*, *y* ∈ *L*,
14. *μ*(*x*) = *μ*((*x* → *y*) → *x*) for all *x*, *y* ∈ *L*,
15. *μ*(*x*) ≥ *μ*(¬*x* → *x*) for all *x* ∈ *L*,
16. *μ*(*x*) = *μ*(¬*x* → *x*) for all *x* ∈ *L*,
17. *μ*(*x* → *z*) ≥ *μ*(*x* → (¬*z* → *y*))∧*μ*(*y* → *z*) for all *x*, *y*, *z* ∈ *L*,
18. *μ*(*x* → *z*) ≥ *μ*(*x* → (¬*z* → *z*)) for all *x*, *z* ∈ *L*,
19. *μ*(*x* → *z*) = *μ*(*x* → (¬*z* → *z*)) for all *x*, *z* ∈ *L*.

## 7. Conclusion

In this paper, we present several characterizations of some fuzzy prefilters (filters) in *EQ*-algebras. Using characterizations of these fuzzy prefilters (filters), we mainly consider the relations among special fuzzy prefilters (filters). We find some conditions under which a fuzzy positive implicative prefilter (filter) is equivalent to a fuzzy implicative prefilter (filter). As applications of our obtained results, we give some new characterizations about classical filters in *EQ*-algebras and some related results about fuzzy filters in residuated lattices. In our future work, we will introduce the notion of states on *EQ*-algebras and discuss the relations between fantastic filters and states on *EQ*-algebras.

## Figures and Tables

**Table 1:** 

⊙	0	*a*	*b*	1

0	0	0	0	0
*a*	0	0	0	*a*
*b*	0	0	0	*b*
1	0	*a*	*b*	1

∼	0	*a*	*b*	1

0	1	*a*	*a*	*a*
*a*	*a*	1	*b*	*b*
*b*	*a*	*b*	1	1
1	*a*	*b*	1	1

→	0	*a*	*b*	1

0	1	1	1	1
*a*	*a*	1	1	1
*b*	*a*	*b*	1	1
1	*a*	*b*	1	1

**Table 2:** 

⊙	0	*a*	*b*	1

0	0	0	0	0
*a*	0	*a*	*a*	*a*
*b*	0	*a*	*b*	*b*
1	0	*a*	*b*	1

∼	0	*a*	*b*	1

0	1	0	0	0
*a*	0	1	*a*	*a*
*b*	0	*a*	1	1
1	0	*a*	1	1

→	0	*a*	*b*	1

0	1	1	1	1
*a*	0	1	1	1
*b*	0	*a*	1	1
1	0	*a*	1	1

**Table 3:** 

⊙	0	*a*	*b*	*c*	1

0	0	0	0	0	0
*a*	0	0	0	0	*a*
*b*	0	0	0	0	*b*
*c*	0	0	0	0	*c*
1	0	*a*	*b*	*c*	1

∼	0	*a*	*b*	*c*	1

0	1	0	0	0	0
*a*	0	1	*b*	*b*	*b*
*b*	0	*b*	1	*c*	*c*
*c*	0	*b*	*c*	1	1
1	0	*b*	*c*	1	1

→	0	*a*	*b*	*c*	1

0	1	1	1	1	1
*a*	0	1	1	1	1
*b*	0	*b*	1	1	1
*c*	0	*b*	*c*	1	1
1	0	*b*	*c*	1	1

**Table 4:** 

⊙	0	*a*	*b*	1

0	0	0	0	0
*a*	0	0	0	*a*
*b*	0	0	*a*	*b*
1	0	*a*	*b*	1

∼	0	*a*	*b*	1

0	1	*b*	*a*	0
*a*	*b*	1	*b*	*a*
*b*	*a*	*b*	1	*b*
1	0	*a*	*b*	1

→	0	*a*	*b*	1

0	1	1	1	1
*a*	*b*	1	1	1
*b*	*a*	*b*	1	1
1	0	*a*	*b*	1
